# Convergence of the Laplace and the alternative multipole expansion approximation series for the Coulomb potential

**DOI:** 10.1038/s41598-023-42724-8

**Published:** 2023-09-25

**Authors:** E. O. Jobunga, C. O. Wandera, O. S. Okeyo

**Affiliations:** 1 Department of Mathematics and Physics, Technical University of Mombasa, P. O. Box 90420-80100, Mombasa, Kenya; 2 Department of Physics and Materials Science, Maseno University, Private Bag-40105, Maseno, Kenya

**Keywords:** Chemistry, Materials science, Mathematics and computing, Physics

## Abstract

Multipole expansion is a powerful technique used in many-body physics to solve dynamical problems involving correlated interactions between constituent particles. The Laplace multipole expansion series of the Coulomb potential is well established in literature. We compare its convergence with our recently developed perturbative and analytical alternative multipole expansion series of the Coulomb potential. In our working, we confirm that the Laplace and the analytical alternative multipole expansion series are equivalent as expected. In terms of performance, the perturbative alternative multipole expansion series underapproximate the expected results to some extent while the Laplace and the analytical alternative multipole expansion series yield results which are relatively accurate but oscillatory in nature even with a higher number of angular momentum terms employed. As a practical example, we have evaluated the Slater double integrals for two-electron systems using the multipole expansion techniques and a mean field approximation. The estimated results show that only spherical interactions are dominant while the higher-order interactions are negligible. To highlight the discrepancy in the application of each of the formulations of the multipole expansion series for the electron-electron interaction potential, an estimation of the non-relativistic groundstate energies of some helium-like systems, evaluated using the spherical approximation of the multipole potential, is provided. Our findings are likely to be useful in the treatment of the Coulomb potential in electronic structure calculations as well as in celestial mechanics.

## Introduction

The Laplace multipole expansion series is established in the works of Laplace and Legendre in their search for solutions to the problem of attractions. The historical developments that led to the derivation of the expansion series and the introduction of the Legendre polynomials, for the first time, as the coefficients used in the Laplace expansion are captured in Laden’s thesis^1^. The Laplace multipole expansion has become conventional knowledge in physics textbooks^2^ and it is quite useful in solving the many-body physics problems in celestial mechanics, quantum physics and chemistry, nuclear physics, and condensed matter physics.

Naturally, the multipole expansion becomes convenient to use in solving physical problems in 3D if expressed in the spherical polar coordinates. This decomposes the problem as a product of both radial and angular parts. The radial part can be treated as a 1D case while the well defined angular momentum algebra^3^ can be used to simplify the angular parts. Several studies have employed multipole expansion techniques in the recent past in solving physical problems of interest^4–9^.

In our alternative multipole expansion of the Coulomb potential^10,11^, we stated that the Laplace multipole expansion series of the Coulomb repulsion term is incomplete, and therefore inaccurate. Vaman clarified that both the Laplace and the alternative multipole expansion are indeed equivalent^12^. Since the Laplace expansion series is a single-index summation while the alternative method is a double-index summation series, it becomes necessary to test the conditions for convergence of the two methods. We also compare the accuracy of the Laplace expansion method, relative to our perturbative and analytical alternative multipole expansion methods, in estimating the expected results. We have seen in literature that such a comparison, not exactly similar to the current study, is reported in ref.^13,14^. Comparison of different methods allows characterization of relative accuracy and capabilities, which is quite instrumental in guiding application^13^. This is particularly important given the fact that the alternative multipole expansion has already been successfully employed in determining the electronic structure for neutral atoms^15,16^.

## Theory

The Coulomb repulsion potential term can be expressed as

1 $$\begin{aligned} \frac{1}{\mid \vec {r}_i-\vec {r}_j \mid }=\frac{1}{r_>}\,\left( 1-2x{\tilde{t}} + {\tilde{t}}^2 \right) ^{-\frac{1}{2}} \end{aligned}$$

which reduces to the Laplace multipole expansion series,

2 $$\begin{aligned} \frac{1}{\mid \vec {r}_i-\vec {r}_j \mid } =\frac{1}{r_>}\,\sum _{l=0}^{\infty }{\tilde{t}}^l\, P_l(x), \end{aligned}$$

where ${{\tilde{t}}=r_</r_>}$, ${r_> = {\max}\{r_i, r_j\}}$, ${r_< = {\min}\{r_i, r_j\}}$, ${x= \cos \theta }$, with ${\theta }$ being the relative angle between the position vectors ${\vec {r}_i}$ and ${\vec {r}_j}$, *l* are non-negative integers, and ${P_l(x)}$ are the $l{\textrm{th}}$ order Laplace coefficients of ${{\tilde{t}}^l}$, also known as the Legendre polynomials. It is important to note that the form given by Eq. (1) is considered as the generating function for the Legendre polynomials^2,17^.

In the alternative approach^10,11^, the multipole expansion of the Coulomb potential

3 $$\begin{aligned} \frac{1}{\mid \vec {r}_i-\vec {r}_j \mid } =\frac{1}{r_>}\,\sum _{l=0}^{\infty } h_l({\tilde{t}})\,P_l(x) \end{aligned}$$

can also be expressed in the basis of Legendre polynomials, where the coefficients

4 $$\begin{aligned} h_l({\tilde{t}}) = \frac{(2l+1)}{\sqrt{1+{\tilde{t}}^2}}{\tilde{j}}_l({\tilde{t}}), \end{aligned}$$

are a function of the spherical Bessel-like functions, ${{\tilde{j}}_l({\tilde{t}})}$, which can be expanded in the perturbative polynomial form as^10,11^

5 $$\begin{aligned} j_0 ({\tilde{t}})= & {} 1 +\sum \limits _{k=1}^\infty \frac{(4k-1)!!}{(2k)!!(2k+1)!!} \left( \frac{{\tilde{t}}}{1+{\tilde{t}}^2}\right) ^{2k} \end{aligned}$$

6 $$\begin{aligned} j_{l>0} ({\tilde{t}})= & {} \sum \limits _{k=0}^\infty \frac{(2l+4k-1)!!}{(2k)!!(2l+2k+1)!!} \left( \frac{{\tilde{t}}}{1+{\tilde{t}}^2}\right) ^{l+2k} \end{aligned}$$

or analytically as a differential equation^11^

7 $$\begin{aligned} {\tilde{j}}_l(t) = (-1)^l \frac{t^{l}}{(2l+1)!!}\left[ \frac{1}{t} \frac{\textrm{d}}{\textrm{dt}} \right] ^l f(t), \end{aligned}$$

with

8 $$\begin{aligned} f(t) = \left\{ \frac{1}{2t}\left[ ({1+2t})^{l+\frac{1}{2}} - ({1-2t})^{l + \frac{1}{2}}\right] \right\} \end{aligned}$$

and

9 $$\begin{aligned} t = \frac{r_i\,r_j}{r_i^2 + r_j^2} = \frac{{\tilde{t}}}{1+{\tilde{t}}^2} \end{aligned}$$

defined in terms of ${\tilde{t}}$ in this case.

The equivalence of Eqs. (2) and (3) shows that

10 $$\begin{aligned} {\tilde{t}}^l = \frac{(2l+1)}{\sqrt{1+{\tilde{t}}^2}}\sum _{k=0}^{k_{\max} \rightarrow \infty } \frac{(2l+4k-1)!!}{(2k)!!(2l+2k+1)!!} \left( \frac{{\tilde{t}}}{1+{\tilde{t}}^2}\right) ^{l+2k} \end{aligned}$$

is an identity.

From the identity relation in Eq. (10), we can further infer that:

11 $$\begin{aligned}&\sum \limits _{k=0}^{k_{\max}\rightarrow \infty } \frac{(2l+4k-1)!!}{(2k)!!(2l+2k+1)!!} \left( \frac{{\tilde{t}}}{1+{\tilde{t}}^2}\right) ^{2k} = \frac{(1+{\tilde{t}}^2)^{l + \frac{1}{2}}}{2l+1}, \end{aligned}$$

12 $$\tilde{j}_{l} (\tilde{t}) = \frac{{\tilde{t}^{l} }}{{2l + 1}}\sqrt {1 + \tilde{t}^{2} } ,$$

13 $$\begin{aligned}&{\tilde{j}}_l({\tilde{t}}) = \left( \frac{2l-1}{2l+1}\right) {\tilde{t}}\,{\tilde{j}}_{l-1}({\tilde{t}}) \end{aligned}$$

14 $$\begin{aligned}&{\tilde{j}}_l({\tilde{t}}) = \frac{{\tilde{t}}^l}{2l+1}\,{\tilde{j}}_0({\tilde{t}}) \end{aligned}$$

Using the relations given by Eq. (9), we have analytically tested and confirmed the inferences given by Eqs. (11)–(12) for the first two orders of the spherical Bessel-like functions herebelow. The zeroth-order spherical Bessel-like function simplifies to:

15 $$\begin{aligned} \begin{aligned} {\tilde{j}}_0({\tilde{t}})&= \frac{\sqrt{1+2t}-\sqrt{1-2t}}{2t} = \frac{\sqrt{1+\frac{2{\tilde{t}}}{1+{\tilde{t}}^2}}-\sqrt{1-\frac{2{\tilde{t}}}{1+{\tilde{t}}^2}}}{\frac{2{\tilde{t}}}{1+{\tilde{t}}^2}} = \frac{\sqrt{1+{\tilde{t}}^2} \left[ \sqrt{1+2{\tilde{t}}+ {\tilde{t}}^2}-\sqrt{1-2{\tilde{t}}+ {\tilde{t}}^2}\right] }{2{\tilde{t}}} \\&= \frac{\sqrt{1+{\tilde{t}}^2} \left[ (1+{\tilde{t}})-(1-{\tilde{t}})\right] }{2{\tilde{t}}} = \sqrt{1+{\tilde{t}}^2} \end{aligned} \end{aligned}$$

Likewise, the first-order spherical Bessel-like function simplifies to:

16 $$\begin{aligned} \begin{aligned} {\tilde{j}}_1({\tilde{t}})&=-\frac{1}{3!!}\frac{d}{dt}\left\{ \frac{1}{2t}\left[ (1+2t)^\frac{3}{2} - (1-2t)^\frac{3}{2}\right] \right\} = \frac{1}{6t^2}\left[ (1+2t)^\frac{3}{2} - (1-2t)^\frac{3}{2}\right] - \frac{1}{2t}\left[ (1+2t)^\frac{1}{2} + (1-2t)^\frac{1}{2}\right] \\&= \frac{\sqrt{1+{\tilde{t}}^2}}{6{\tilde{t}}^2}\left[ (1+{\tilde{t}})^3-(1-{\tilde{t}})^3\right] - \frac{\sqrt{1+{\tilde{t}}^2}}{2{\tilde{t}}}\left[ (1+{\tilde{t}})+(1-{\tilde{t}})\right] \\&= \sqrt{1+{\tilde{t}}^2} \left[ \frac{1}{{\tilde{t}}} + \frac{{\tilde{t}}}{3} -\frac{1}{{\tilde{t}}} \right] = \frac{{\tilde{t}}}{3}\sqrt{1+{\tilde{t}}^2}. \end{aligned} \end{aligned}$$

The use of the recurrence relations given by Eqs. (13) and (14) can be useful in eliminating singularities associated with the analytical expression of the spherical Bessel-like functions, ${\tilde{j}}_l({\tilde{t}})$^11^, as ${{\tilde{t}}\rightarrow 0}$.

As a practical example, we use the Laplace and the alternative multipole expansion series, within a meanfield approximation, to estimate the Slater double integrals $F^l$^18^

17 $$F_{{1s,nl}}^{l} (r_{i} ,r_{j} ) = \left\langle {\tilde{t}} \right\rangle ^{l} \int_{0}^{\infty }dr_{i} {r_{i}^{2} } R_{{n,l}} (r_{i} )R_{{n^{\prime } ,l^{\prime } }} (r_{i} )\left[ {\frac{1}{{r_{i} }}\int_{0}^{{r_{i} }} dr_{j}{r_{j}^{2} } R_{{n^{\prime } ,l^{\prime } }} (r_{j} )R_{{n,l}} (r_{j} ) + \int_{{r_{i} }}^{\infty }dr_{j} {r_{j} } R_{{n^{\prime } ,l^{\prime } }} (r_{j} )R_{{n,l}} (r_{j} )} \right]$$

for helium-like systems, where the higher-order terms involve the exchange of angular momentum quantum number between the *s* and the ${l^{\textrm{th}}}$ orbital. The optimization is based on the root mean value of *t*,

18 $$\begin{aligned} \langle t \rangle = \left\langle \frac{ {\tilde{t}}}{1+ {\tilde{t}}^2 } \right\rangle = \frac{1}{4\pi \sqrt{2}}, \end{aligned}$$

per solid angle, obtained by determining the root mean square value of *t* scaled by $2\pi$ as given by Eq. (32) of ref.^16^. This, consequently, yields

19 $$\begin{aligned} \langle {\tilde{t}} \rangle = 5.6426216557 \times 10^{-2}. \end{aligned}$$

The unscreened hydrogenic radial orbitals can be employed as the trial wavefunctions in evaluating Eq. (17).

In solving the Schrödinger equation for the two-electron atomic systems,

20 $$\begin{aligned} h_i | \phi (r_i) \rangle = \epsilon _{\alpha _i} | \phi (r_i) \rangle , \end{aligned}$$

with single-electron wavefunction $\phi (r_i)$ and the orbital energy eigenvalues $\epsilon _{\alpha _i}$, the single-electron Hamiltonian operator (in atomic units) can be expressed as

21 $$\begin{aligned} h_i = \frac{p_i^2}{2} - \frac{Z}{r_i} + \gamma _{l_i} \,\frac{1}{r_{ij}} \end{aligned}$$

where ${\gamma _{l_i}=\frac{1}{2}}$ is the orbital angular momentum-dependent partitioning fraction^15,16^ for the systems in their groundstate. The first, second, and third terms in Eq. (21) are the kinetic energy term, the electron-nuclear interaction, with *Z* as the nuclear charge, and the electron-electron interaction respectively. The second and the third terms form the potential energy function for the systems.

Within the spherical approximation, the potential energy functions for the system reduce to

22 $$\begin{aligned} V(r_i,r_j) = - \frac{Z}{r_i} + \gamma _{l_i} \,\frac{1}{r_>} \end{aligned}$$

using the Laplace multipole expansion series, or to

23 $$\begin{aligned} V(r_i,r_j) = - \frac{Z}{r_i} + \gamma _{l_i} \,\frac{{\tilde{j}}_0(t)}{\sqrt{r_i^2+r_j^2}} \end{aligned}$$

using the alternative multipole expansion series.

Making use of the mean field approximation given by Eq. (18), and the minimization of the lowest-order term of the multipole potential energy function, as derived in refs.^15,16^, Eq. (23) can be re-written as a single-electron potential

24 $$\begin{aligned} V(r_i) = - \frac{Z_{\textrm{eff}}}{r_i} \end{aligned}$$

with an effective nuclear charge

25 $$\begin{aligned} Z_{\textrm{eff}} = Z- \gamma _{l_i}\,\langle {\tilde{j}}_0(t)\rangle \root 3 \of {\frac{Z}{\gamma _{l_i}}}. \end{aligned}$$

The second term of Eq. (25) can be seen as a nuclear charge screening parameter which is dependent on the magnitude of nuclear charge and the partitioning fraction.

Likewise, Eq. (22) can be expressed as

26 $$\begin{aligned} V(r_i) = - \frac{Z}{r_i} + \gamma _{l_i} \, \left\langle \frac{1}{r_>} \right\rangle \end{aligned}$$

with

27 $$\begin{aligned} \left\langle \frac{1}{r_>} \right\rangle = \frac{1}{r_i}\int _0^{r_i}r_j^2\, dr_j\, R_{n',l'}(r_j)R_{n,l}(r_j) + \int _{r_i}^{\infty }r_j\, dr_j\, R_{n',l'}(r_j)R_{n,l}(r_j) \end{aligned}$$

expressed in terms of the spatial coordinate, $r_i$, using the Slater integral as defined in Eq. (17) or probabilistically as

28 $$\begin{aligned} \left\langle \frac{1}{r_>} \right\rangle = \frac{1}{2}\left[ \frac{1}{r_i} + \left\langle \frac{1}{r_j}\right\rangle \right] = \frac{1}{2}\left[ \frac{1}{r_i} + Z \right] \end{aligned}$$

assuming that $r_i$ and $r_j$ have an equal probability of being greater. Other possible assumed scenarios can include ${r_i \le r_j}$ or ${r_j \le r_i}$.

For the alternative multipole expansion method, the energy eigenvalue

29 $$\begin{aligned} \epsilon _{\alpha _i}^{A} = -\frac{Z_{\textrm{eff}}^2}{2n_i^2}, \end{aligned}$$

for an orbital with the principal quantum number $n_i$, can be obtained directly by using the effective potential defined in Eq. (25). However, for the Laplace multipole expansion method, the Schrödinger equation must be solved numerically using the potential defined by Eqs. (26) and (27) or perturbatively

30 $$\begin{aligned} \epsilon _{\alpha _i}^{L} = -\frac{{\tilde{Z}}_{\textrm{eff}}^2}{2n_i^2} + \frac{1}{2}\gamma _{l_i}\,Z \end{aligned}$$

using

31 $$\begin{aligned} {\tilde{Z}}_{\textrm{eff}} = Z-\frac{1}{2}\gamma _{l_i} \end{aligned}$$

as the adjusted effective charge corresponding to the scenario assumed in Eq. (28).

With the energy eigenvalues, the non-relativistic groundstate energy for two-electron atomic systems, with $1s^2$ configuration, can be determined by^16^

32 $$\begin{aligned} E_{1s-1s} = 4 \times \epsilon _{1s}, \end{aligned}$$

employing the anti-symmetric indistinguishable Hartree-Fock wavefunctions

33 $$\begin{aligned} \Psi _{\alpha _i,\alpha _j} (\vec {q}_i,\vec {q}_j)= \frac{1}{\sqrt{2}}\left[ \phi _{\alpha _i} (\vec {q}_i)\phi _{\alpha _j} (\vec {q}_j)- \phi _{\alpha _i} (\vec {q}_j)\phi _{\alpha _j} (\vec {q}_i)\right] , \end{aligned}$$

with the spatial and spin coordinates ${\vec {q}_i = (\vec {r}_i, \vec {s}_i)}$, in the solution of the Schrödinger equation for the separable Hamiltonian derived using the multipole expansion series.

## Results

Our goal in this work is to test the convergence of the Laplace and the alternative multipole expansion series and also to compare the performance of both methods in estimating the exact function given by Eq. (1). The spherical Bessel-like functions, ${\tilde{j}}_l({\tilde{t}})$, used in the alternative multipole expansion can be evaluated perturbatively as given by Eqs. (5) and (6) or analytically as given by Eq. (7). Our calculations for convergence and performance are computed both perturbatively and analytically. As an example to test the convergence of the two approaches in real physical applications, we have calculated the Slater integrals and the non-relativistic groundstate energy of two-electron helium-like systems within the spherical approximation of the electron-electron interaction.

**Figure 1:**
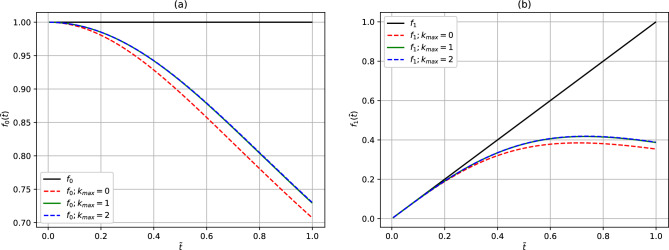
(Color online) Comparison of the functions (**a**) ${f_0({\tilde{t}})=1}$ and ${h_0({\tilde{t}})=f_0^{k_{\max}}({\tilde{t}})}$ and (**b**) ${f_1({\tilde{t}})={\tilde{t}}}$ and ${h_1({\tilde{t}})=f_1^{k_{\max}}({\tilde{t}})}$ , summed up to the maximum value ($k_{\max}$), plotted using left and right hand side of Eq. (10) respectively. The black solid line corresponds to the Laplace basis functions, ${{\tilde{t}}^l}$.

In Fig. 1, we plot the convergence of the first two orders of the Laplace functions, ${\tilde{t}}^l$, relative to the alternative multipole expansion functions, ${h_l({\tilde{t}})}$, as given by Eq. (10). The domain ${0 \le {\tilde{t}} \le 1}$ has been chosen to coincide with the regime of convergence of the Laplace multipole expansion series. The convergence tests should confirm the validity of the identity relations given by the stated equation. Since ${h_l({\tilde{t}})}$ is an infinite series function, it can be seen that only three terms (with ${k_{\max}=2}$) of the summation series already yield reasonable trend of convergence, albeit slowly. In subsequent figures, we use ${h_l^{k_{\max}=2}({\tilde{t}})}$ as our best converged perturbative results. From Eqs. (4)–(6), it can be seen that the divergence between the Laplace functions and the perturbative alternative multipole expansions stems from the approximation of the factor ${\sqrt{1+{\tilde{t}}^2} \rightarrow 1}$ as ${k_{\max}\rightarrow 0}$.

**Figure 2:**
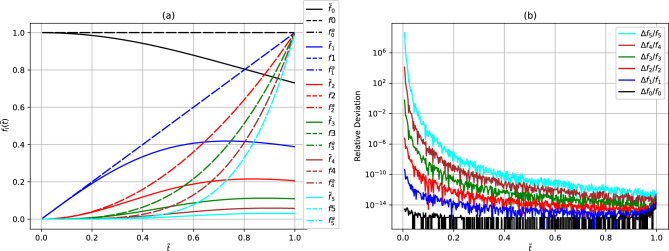
(Color online) (**a**) Comparison of the six functions of ${f_l({\tilde{t}})=t^l}$, $h_l({\tilde{t}})={\tilde{f}}_l^{k_{\max}}({\tilde{t}})$ with the value ${k_{\max}=2}$, and the analytical $h_l({\tilde{t}})={\tilde{f}}_l({\tilde{t}})$, plotted using left and right hand side of Eq. (12) respectively. The solid and the dash-dot lines represent the perturbative and the analytical $h_l({\tilde{t}})$ functions, as given by Eqs. (5)–(7), while the dashed lines represent the Laplace basis functions, $f_l({\tilde{t}})={\tilde{t}}^l$, respectively. The analytical $h_l({\tilde{t}})$ functions and the Laplace basis functions are overlapping in the figure. (**b**) The relative deviation given as the absolute difference between the analytical $h_l({\tilde{t}})$ and the Laplace $f_l({\tilde{t}})={\tilde{t}}^l$ functions divided by the Laplace functions.

In Fig. 2a, we compare the convergence of the perturbative results with the corresponding analytical, ${h_l({\tilde{t}})}$, functions and the Laplace basis functions as given by Eqs. (10) and (12) for the first six orders of *l*. As already shown in Fig. 1, except at lower values of ${{\tilde{t}}}$, the perturbative basis functions do not agree fully with the corresponding Laplace basis functions in all the cases considered. As expected, the analytical basis functions, on the other hand, show an excellent agreement with the corresponding Laplace basis functions. In the figure, the analytical $h_l({\tilde{t}})$ functions and the Laplace basis functions are overlapping in the entire domain. In Fig. 2b, we show the relative deviation between the analytical and the Laplace basis functions. The relative deviations are calculated as the absolute difference between the analytical $h_l({\tilde{t}})$ and the Laplace $f_l({\tilde{t}})={\tilde{t}}^l$ functions divided by the Laplace functions. The observed relative deviations can be attributed to numerical noise as well as to the divergences due to singularities in the analytical function as ${{\tilde{t}}} \rightarrow 0$.

**Figure 3:**
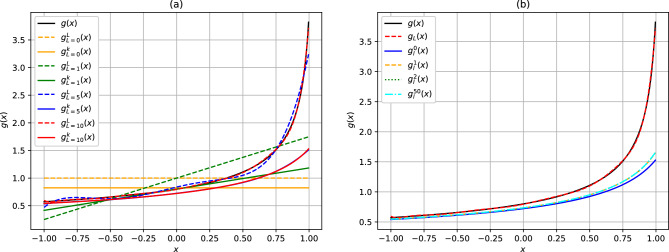
(Color online) Convergence of the Laplace and the perturbative alternative multipole expansion series in comparison to the expected function ${g (x, {\tilde{t}}) = (1-2x{\tilde{t}} + {\tilde{t}}^2 )^{-\frac{1}{2}}}$ given by Eq. (1) , at ${{\tilde{t}}=0.75}$, as a function of: (**a**) the angular momenta *L* with ${k_{\max}=2}$ and, (**b**) ${k_{\max}}$ with ${L_{\max}=10}$. The black solid line is the expected curve. The blue and red solid lines in (**a**) are overlapping. The Laplace functions are denoted by dashed lines in (**a**) and $g_L$ in (**b**).

**Figure 4:**
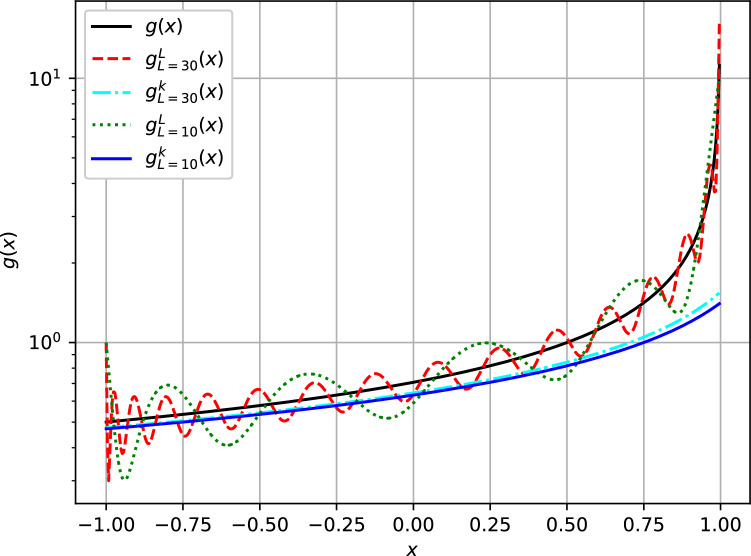
(Color online) Convergence of the Laplace and the perturbative alternative multipole expansion series in comparison to the expected function ${g (x, {\tilde{t}}) = (1-2x{\tilde{t}} + {\tilde{t}}^2 )^{-\frac{1}{2}}}$ given by Eq. (1) , at ${{\tilde{t}}=1.00}$, as a function of the angular momenta (${L_{\max}=10}$ and ${L_{\max}=30}$) with ${k_{\max}=2}$. The black solid line is the expected curve. The Laplace functions are denoted by $g_L^L$ while the perturbative function by $g_L^k$. The logarithmic scale has been used for clarity.

Because of the slow convergence of the perturbative functions, it became of importance to test the performance of the expansions in approximating the value of the analytic function given by Eq. (1) for various values of ${{\tilde{t}}}$ across the angular spectrum. The performance results are summarized in Fig. 3 for all values of ${x= \cos \theta }$. For lower values of ${{\tilde{t}}}$, fewer angular momentum values are necessary for convergence. For ${{\tilde{t}}=0.75}$, reasonable convergence is obtained with ${l_{\max}=10}$. The perturbative expansion on the other hand converges faster with fewer values of ${l_{\max}}$ and ${k_{\max}}$, although the expected results are underapproximated to some extent using this approximation. In particular, complete convergence for the perturbative expansion is obtained using ${l_{\max}=5}$ and ${k_{\max}=2}$ only. As ${{\tilde{t}}\rightarrow 1}$, a higher number of angular momenta are necessary for convergence if the Laplace or the analytical multipole functions are used. In Fig. 4, we show that for ${{\tilde{t}}= 1}$ convergence of the expected function is not yet achieved even with ${l_{\max}=30}$ for the Laplace and the analytical multipole expansion. It can also be observed in Fig. 4 that as the angular momenta increases, the period and the amplitude of oscillation of the Laplace and the analytical multipole expansion results reduces. The perturbative expansion, on the other hand, is converged with less angular momenta and shows remarkable stability in the approximation of the expected function. The perturbative results offer the possibility to isolate features that are dependent on the lower order terms of the multipole expansion of the Coulomb potential.

The equivalence between the Laplace and the analytical alternative multipole expansion methods provides a wider choice of techniques to use when dealing with the Coulomb repulsion term. The Laplace basis functions appear simpler, in comparison with the analytical functions, but the underlying difficulty lies in the uncertainty of identifying the ${r_>}$ and ${r_<}$ variables. In the analytical alternative multipole expansion, on the other hand, it is not necessary to identify the ${r_>}$ and ${r_<}$ variables in the mathematical manipulation of the electron-electron interaction. Additionally, as already shown in references^15,16^, the correlated term becomes separable in the alternative multipole expansion within some meanfield approximation, yielding a definite nuclear charge screening parameter, making it quite favourable to use for computations.

As a practical example, we have compared the Slater integrals^18^ for the $1s-nl$ interacting states estimated using the Laplace and the lowest-order perturbative alternative multipole expansion series for two electron systems as expressed in Eq. (17). The results are presented in Table 1. The calculations have been done using the unscreened hydrogenic radial wavefunctions and the mean-field approximation in Eq. (19). As expected the analytical alternative multipole expansion yields results equivalent to the Laplace multipole expansion results. The lowest-order perturbative alternative multipole expansion results, on the other hand, are slightly less by a factor. From the results presented in the table, it is evident that the higher order multipole interactions are negligible and only become important when the lower -order interactions vanish.

**Table 1:** Comparison of Slater integrals for the $1s-nl$ interacting states evaluated using Eqs. (17) and (19) for the Laplace and the lowest-order perturbative alternative multipole expansion of the Coulomb repulsion term. The calculations have been done using the unscreened hydrogenic radial wavefunctions.

$F^l$	$1s-nl$	Laplace	Perturbative
$F^0$	1s-1s	$0.6250\,Z$	$0.6240\,Z$
$F^1$	1s-2p	$4.5409 \times 10^{-3}\,Z$	$4.5337 \times 10^{-3}\,Z$
$F^2$	1s-3d	$8.8323 \times 10^{-6}\,Z$	$8.8183 \times 10^{-6}\,Z$

As a test of convergence in the application of both multipole series expansion methods, we have estimated the non-relativistic groundstate energies of helium-like systems, with ${1\le Z \le 10}$, using the spherical approximation of the series. For ease of comparison, we have generated separable interaction potentials corresponding to both expansion series. Making the Laplace potential separable is quite a challenging task though. To achieve this goal, we have created three different scenarios representing all possible configurations in the electronic distributions. Firstly, we have assumed that the spatial coordinate ${r_i}$ is greater that the other coordinate ${r_j}$ in the entire domain. Secondly, we assumed that the reverse is true (${r_j>r_i}$), and lastly we have assumed that either ${r_i}$ or ${r_j}$ has an equal probability of being greater. With separable potentials, it is possible to solve the Schrödinger equation analytically for the two-electron systems. To enhance clarity in our comparison, we use the Hartree-Fock expansion of the wavefunction for all the cases considered. We have also avoided the use of self consistent calculations, which usually require several iterations for convergence, by choosing to use the separable potentials. The results of our estimation are presented in Table 2 in comparison with the literature values^19^.

From the results in Table 2, it can be seen that, except for the negatively charged hydrogen ion which has varied results, the Laplace multipole expansion approximation yields tight binding groundstate energies as presented in the three scenarios. The scenario with ${r_i \le r_j}$ have the highest binding energies while the scenario with ${r_j \le r_i}$ have the least among the three scenarios presented. The correlation between the scenarios is also evident, that is, the corresponding results have a common difference among the positively charged ions. Using the literature values^19^ as our benchmark, we can say that Lap$^1$ results are the best case scenario for the Laplace multipole expansion for two-electron systems since the groundstate energy of the negatively charged hydrogen ion is quite close to the reference value. The effect of the electron-electron interaction for the Laplace scenarios can be seen to be a weak perturbation at higher *Z* values. This is because the nuclear charge screening term appears to be independent of the nuclear charge.

The alternative multipole approximation results in Table 2, on the other hand, presents the least binding energies for the positive charged ions. This is because of the optimization used in generating the separable potential, which associates the nuclear charge screening parameter with the nuclear charge itself and in the process, maximising the electron-electron interaction. However, it can be observed that, except for the negatively charged hydrogen ion, the binding energies for the alternative multipole expansion approximation are still higher than the literature values^19^. The disparity with literature values widens with increase in charge polarisation, ${Z_p=Z-Z_e}$, with *Z* as the nuclear charge and $Z_e$ as the total electronic charge. This could be a pointer to a possible inadequacy of the Hamiltonian used in accounting for some of the interactions. Future investigations can probe the disparity further, for example, by including the higher-order multipole interactions as well as the relativistic effects like spin-orbit coupling and spin-spin interactions.

**Table 2:** Comparison of the non-relativistic groundstate $(1s^2)$ energies of helium-like systems with ${1\le Z\le 10}$ estimated using Eqs. (22) and (23) for the Laplace and the alternative forms of multipole expansion of the Coulomb repulsion term respectively.

*Z*	Lap.$^1$	Lap.$^2$	Lap.$^3$	Alt.	Ref.
1	− 0.500	− 0.0	− 0.125	− 0.27238	− 0.4722
2	− 4.500	− 4.0	− 4.125	− 2.90422	− 2.9035
3	− 12.500	− 12.0	− 12.125	− 8.73614	− 7.2797
4	− 24.500	− 24.0	− 24.125	− 17.9809	− 13.6556
5	− 40.500	− 40.0	− 40.125	− 30.7495	− 26.9091
6	− 60.500	− 60.0	− 60.125	− 47.1122	− 38.2881
7	− 84.500	− 84.0	− 84.125	− 67.1179	− 51.6698
8	− 112.500	− 112.0	− 112.125	− 90.8033	− 67.0551
9	− 144.500	− 144.0	− 144.125	− 118.197	− 84.4451
10	− 180.500	− 180.0	− 180.125	− 149.321	− 103.843

Lap.$^1$ and Lap.$^2$ results have been evaluated using Eq. (22) assuming that ${r_> = r_i}$ and ${r_> = r_j}$ respectively, while Lap.$^3$ results have been evaluated using Eq. (28), considering both possibilities with equal distribution. The calculations have been benchmarked with ref.^19^ data extracted from the provided ionization energies and electron affinities, that is, ${E_{1s-1s}= -\frac{1}{2}\,Z^2 - I_p}$, where $I_p$ is the ionization energy (or electron affinity).

## Conclusion

The convergence as well as the performance of the Laplace multipole expansion of the Coulomb potential, in comparison with our recently developed alternative multipole expansion series, is investigated in this study. We have confirmed that the Laplace and the analytical alternative multipole expansion series are indeed equivalent and offer a higher degree of accuracy if a larger $l_{\max}$ is used in the approximation. The perturbative alternative multipole expansion, on the other hand, converges with a much lower value of $l_{\max}$ and $k_{\max}$ and is stable against oscillations in results as ${{\tilde{t}}\rightarrow 1}$ but the converged results underapproximate the expected results to some extent at all angles. The stability of the perturbative results may be useful in isolating physically meaningful features even with less angular momenta in converged results.

As a practical example, we have shown that, despite the equivalence of the two formulations of the multipole expansion series, the results generated by each form can be very different depending on the optimization procedures used. In the present case, in the solution of the non-relativistic groundstate energy for helium-like systems with ${1\le Z\le 10}$, the alternative multipole expansion leads to an effective nuclear charge with a charge-dependent nuclear screening parameter. The Laplace multipole expansion, on the other hand, leads to a nuclear screening parameter which is charge independent. The disparity in the results obtained in the two approaches is large. Comparatively, the alternative multipole expansion performs better relative to the literature values, with the disparity in results varying as a function of the charge polarisation between the nucleus and electrons.

## Data Availability

All the data generated in the work are embedded as figures in the manuscript.
